# Training health-care practitioners in screening, brief intervention, and referral to treatment using standardized approaches and expert coaching

**DOI:** 10.1186/1940-0640-7-S1-A95

**Published:** 2012-10-09

**Authors:** Mary Velasquez, Sylvia Shellenberger, Kirk von Sternberg

**Affiliations:** 1 School of Social Work, University of Texas, Austin, TX, USA; 2 Department of Family Medicine, Mercer University School of Medicine, Macon, GA, USA

## 

Skill acquisition and maintaining fidelity to a practice requires ongoing monitoring and performance feedback in addition to initial training. This paper describes an evaluation of a training and coaching model employed for the Texas and Georgia screening, brief intervention, and referral to treatment (SBIRT) programs, funded by the US Substance Abuse and Mental Health Services Administration. Both programs were both implemented in large community medical settings that included hospital emergency departments, trauma and inpatient services, and primary care. We describe the specialist training processes with an emphasis on our web- and phone-based expert coaching model and standardized training programs. Specialists who received the multifaceted training package demonstrated significant improvements in SBIRT skills during a half-day standardized patient activity and over 12 months of coaching as measured by the Motivational Interviewing Treatment Integrity (MITI) coding system and coaching evaluations. Videotaped recordings of four standardized patient training sessions for 20 randomly selected trainees were coded by an expert external coder using the MITI-3 coding system. Trainees made significant improvements in evocation (p = 0.004), collaboration (p = 0.003), direction (p = 0.030), empathy (p = 0.029), spirit (p = 0.008), and in percentage of MI-adherent speech (p = 0.035). Further, 148 quarterly reports were examined for those trainees who received at least 12 months of coaching. Thirty-seven trainees (17 from Texas and 20 from Georgia) were included in these analyses. Trainee global ratings on spirit (Texas, p = 0.026; Georgia, p = 0.002) and empathy (Texas, p = 0.012; Georgia, p = 0.001) improved during the four quarters. In addition, MI-behavior ratings for both sites showed improvement at some point over the four quarters analyzed (p < 0.05). This study adds evidence to previous findings indicating that progressive feedback and coaching enhance SBIRT skill development.

